# Causal connectivity alterations of cortical-subcortical circuit anchored on reduced hemodynamic response brain regions in first-episode drug-naïve major depressive disorder

**DOI:** 10.1038/srep21861

**Published:** 2016-02-25

**Authors:** Qing Gao, Ke Zou, Zongling He, Xueli Sun, Huafu Chen

**Affiliations:** 1 School of Mathematical Sciences, University of Electronic Science and Technology of China, Chengdu, 611731, P.R. China; 2 Neurobiological laboratory, West China Hospital of Sichuan University, Chengdu, 610041, P.R. China; 3 Mental Health Center of Chengdu, Chengdu, 610016, P.R. China; 4 Mental Health Center, West China Hospital of Sichuan University, Chengdu, 610041, P.R. China; 5 Center for Information in BioMedicine,Key Laboratory for Neuroinformation of Ministry of Education, School of Life Science and Technology, University of Electronic Science and Technology of China, Chengdu, 610054, P.R. China

## Abstract

Some efforts were done to investigate the disruption of brain causal connectivity networks involved in major depressive disorder (MDD) using Granger causality (GC) analysis. However, the homogenous hemodynamic response function (HRF) assumption over the brain may disturb the inference of temporal precedence. Here we applied a blind deconvolution approach to examine the altered HRF shape in first-episode, drug-naïve MDD patients. The regions with abnormal HRF shape in patients were chosen as seeds to detect the GC alterations in MDD. The results demonstrated significantly decreased magnitude of spontaneous hemodynamic response of the orbital frontal cortex (OFC) and the caudate nucleus (CAU) in MDD comparing to healthy controls, suggesting MDD patients likely had alterations in neurovascular coupling and cerebrovascular physiology in these two regions. GC mapping showed increased/decreased GC in OFC-/CAU centered networks in MDD. The outgoing GC values from OFC to anterior cingulate cortex and occipital regions were positively correlated with Hamilton Depression Scale (HAMD) scores, while the incoming GC from insula, middle and superior temporal gyrus to CAU were negatively correlated with HAMD scores of MDD. The abnormalities of directional connections in the cortico-subcortico-cerebellar network may lead to unbalanced integrating the emotional-related information for MDD, and further exacerbating depressive symptoms.

Increasing neuroimaging evidence has emphasized major depressive disorder (MDD) as a network-level neural disorder, associated with the dysregulation of a distributed brain network emcompassing the cortical-subcortical-cerebellar circuit^1^,^2^,^3^,^4^,^5^. Important progress has been made in understanding the pathogenesis of MDD by investigating the brain’s intrinsic functional connectivity networks of resting-state functional magnetic resonance imaging (fMRI) data^3^,^4^,^6^. Other than functional connectivity method which measures statistical dependencies of time-series between distinct units, effective connectivity or causal connectivity investigates the influence one neuronal system exerts over another^7^. Granger causality (GC) analysis is one of the powerful and widely applicable techniques to detect the effective connectivity between even remote brain regions^8^,^9^. Specifically, GC analysis has recently been increasingly employed in the studies of depression to identify the effective connectivity abnormality in MDD^1^,^2^,^10^,^11^. Using GC analysis, increased excitatory effect from hippocampus to anterior cingulated cortex (ACC), and increased inhibition in activity of dorsal cortical structures by hippocampus and ACC in MDD patients were found^2^. Chosen insula (INS) as a seed region, another study demonstrated a failure of reciprocal influence in INS-centered causal network in MDD^10^. GC analysis was also performed to detect abnormal causality connectivity between seeds with reduced gray matter volume and other brain regions, and unidirectionally affected causal connections driven by the structural deficits within the cortico-limbic-cerebellar circuit were found in MDD^1^. Moreover, conditional Granger causality method, which could distinguish the pseudocausal relationship for three or more time series^8^,^12^, was applied to revealed the abnormal fluctuation of the signals of the depression-associated resting-state networks^11^. The study demonstrated the altered default mode network related dynamic interactions with the ventromedial prefrontal network, the salience network and the fronto-parietal network in depression^11^. These findings advanced the causal topology of the brain functional network, and revealed new insights in discovering the neuropathological mechanisms underlying the depressive symptoms.

However, these researches have inconsistent results in detail. One reason of the inconsistency may due to the method considerations. Firstly, most of the fMRI studies based on GC analysis aforementioned always assumed homogeneous hemodynamic processes over the brain. However, several studies have pointed out that hemodynamic response function (HRF) latency across distinct brain regions is variable, and the homogenous HRF assumption may disturb the inference of temporal precedence^9^,^13^. Recently, a novel blind deconvolution approach for resting-state fMRI data was proposed to reconstruct the HRF at each brain voxel, which made it possible to detect deconvolved blood-oxygenation level-dependent (BOLD) level effective connectivity network^9^. HRF shape was characterized by parameters including response height, time-to-peak and full-width at half-max as potential measures of response magnitude, latency, and duration^9^. Relatively stable distributions of the three parameters over the whole brain were also found in the study, suggesting their capabilities to quantify regional properties of brain in resting-state^9^. In addition, the study demonstrated deconvolution might remove spurious correlations and restore genuine correlations obscured by noise, and consequently increased the detection capacity of GC analysis of fMRI data to neural causality^9^.

Secondly, when coping with multivariate datasets, it is necessary to condition the analysis to other variables in order to distinguish among direct and mediated influences^9^. When applying conditional GC to high dimensional networks such as voxel-wise network from the whole brain, a fully multivariate conditioning could result in computational problems and underestimation of causalities in presence of redundant variables^9^,^14^. In order to overcome these issues, Marinazzo *et al.* has proposed that it can be enough to recover a network eliminating spurious influences by conditioning on a small number of variables which are chosen as the most informative ones for each given candidate driver variable^15^. This partially conditioned GC approach could overcome the computational problems and redundancy curse. This approach was found to lead to results very close to those obtained with a fully multivariate analysis, and even better in the presence of a small number of samples^15^.

Another reason for the inconsistent results may be interpreted by the heterogeneity of samples, such as medication use, illness duration and episodes^1^,^16^. For example, antidepressants could reverse gray matter atrophy in MDD^17^. Gray matter abnormalities were found to be associated with illness duration^18^. The aberrant network topology pattern was associated with the course of depressive episodes^6^. Hence, studies exploring first-episode and drug-naïve patients with MDD may be more reliable in elucidating the early-stage nature of MDD^1^,^16^.

In this work, we used the novel partially conditioned GC approach to investigate the alterative network of directed dynamical influences at deconvolved BOLD level in first-episode, drug-naïve, and relatively short duration MDD patients. Since HRF has been found to be reproducible and consistent across healthy subjects^9^,^19^,^20^, and to be related to key physiological factors including cerebral blood flow and the cerebral metabolic rate of oxygen^9^,^19^,^20^,^21^, we hypothesized that the HRF shape in brain regions associated with emotion-processing circuit in MDD differs from that in healthy controls (HC), and the effective connectivity networks centered at these regions would alter in MDD comparing to HC group.

## Results

### Brain regions with HRF abnormality in MDD

**Figure 1:**
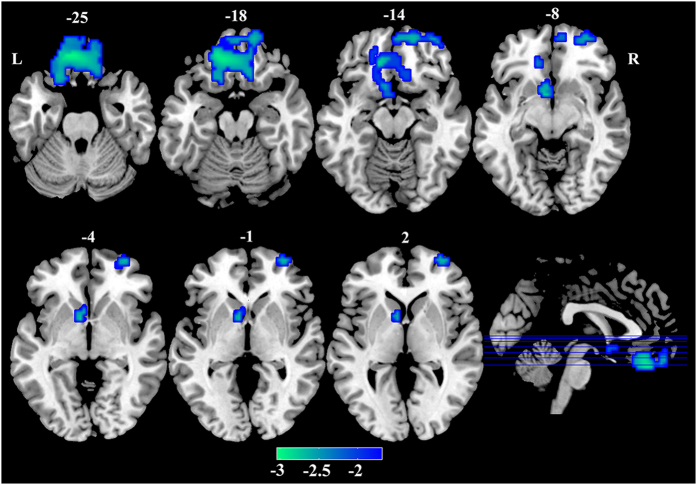
Brain regions with significantly lower response height of HRF in MDD than in HC (*p* < 0.05, AlphaSim corrected).

Only the HRF response height showed the significant difference between MDD and HC groups. Figure 1 demonstrates brain regions with significantly different response height of HRF between the groups (*p* < 0.05, AlphaSim corrected). Compared with HC group, MDD group showed significantly decreased HRF height in the superior frontal gyrus orbital (ORBsup), the middle frontal gyrus orbital (ORBmid) and the inferior frontal gyrus orbital (ORBinf) of the orbital frontal cortex (OFC) extending to the rectus gyrus, and the caudate nucleus (CAU). Significantly higher HRF height in MDD group than HC group was not found in the present study.

Seeds regions of interest (ROI) based on the two sample *t*-test results of the hemodynamic response were selected to calculate the effective connectivity mapping between the seeds regions and the remaining brain regions. They were centered at the peaks *t*-values in left superior frontal gyrus orbital (ORBsup), right middle frontal gyrus orbital (ORBmid) and left inferior frontal gyrus orbital (ORBinf), and left CAU. Table 1 illustrated the details of the chosen seeds, including the Montreal Neurological Institute (MNI) coordinates of peak voxels, cluster sizes and statistical *t*-values.

### Partially conditioned Granger causality mapping of left ORBsup seed

**Figure 2:**
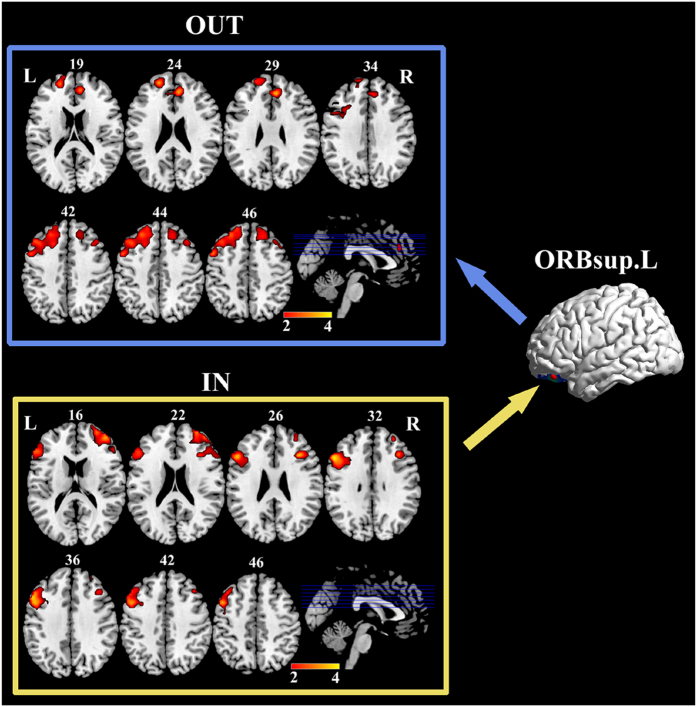
Brain regions with significantly more information transfer from and to left ORBsup in MDD compared with HC. Abbreviations: L = left, ORBsup = superior frontal gyrus orbital, R = right.

Figure 2 demonstrates the brain regions which exhibit significantly different effective connections both to and from left ORBsup in MDD patients comparing to HC group. Table 2 summarized the results in details. In left ORBsup, there is significant stronger causal outflow from left ORBsup to bilateral superior frontal gyrus, middle frontal gyrus and precentral gyrus; and stronger causal inflow from bilateral middle frontal gyrus, inferior frontal gyrus triangular, and right ORBinf to left ORBsup for MDD. No significantly lower information transfer was reported for MDD comparing to HC. The GC values had no significant correlations with illness duration or Hamilton Depression Scale (HAMD) scores.

### Partially Conditioned Granger Causality Mapping of right ORBmid seed

**Figure 3:**
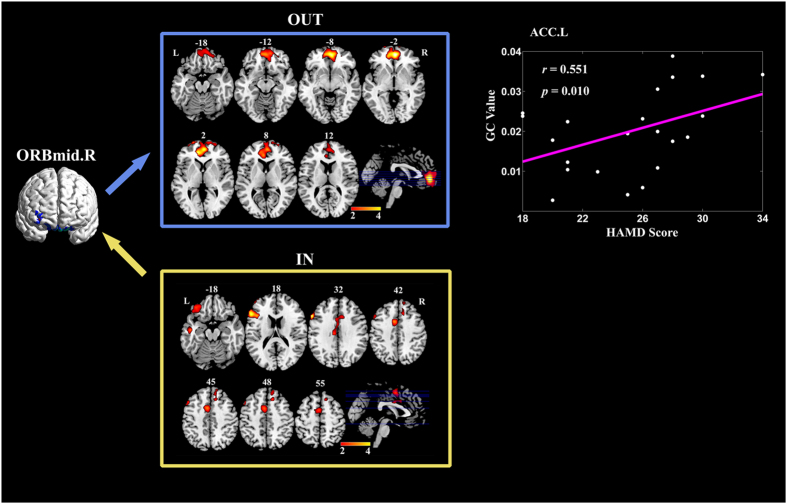
Brain regions with significantly more information transfer from and to right ORBmid in MDD compared with HC. The median correlation coefficient of the significant correlation coefficients between GC values and patients HAMD scores is shown in the most right column. Abbreviations: ACC = anterior cingulate cortex, L = left, ORBmid = middle frontal gyrus orbital, R = right.

The brain regions which exhibit significantly larger effective connections both to and from right ORBmid for MDD are illustrated in Fig. 3. Stronger outgoing information transfer was found from right ORBmid seed to right medial frontal gyrus orbital and left ACC; Stronger incoming information transfer was found from the following regions to the seed: left superior frontal gyrus, left medial frontal gyrus orbital, left inferior frontal gyrus triangular, left Inferior frontal gyrus opercular, left middle temporal gyrus temporal pole and bilateral middle cingulate gyrus (See Table 3 for the details). Specifically, correlation analysis further showed that the GC values from right ORBmid to left ACC were positively correlated with HAMD scores of MDD patients (*p* < 0.05, Alphasim corrected). Table 4 summarized the results of the voxel-wise correlation analysis, including the MNI coordinates of peak voxels, cluster sizes, mean correlation coefficient *r*, std *r* and mean *p* values.

### Partially conditioned Granger causality mapping of left ORBinf seed

**Figure 4:**
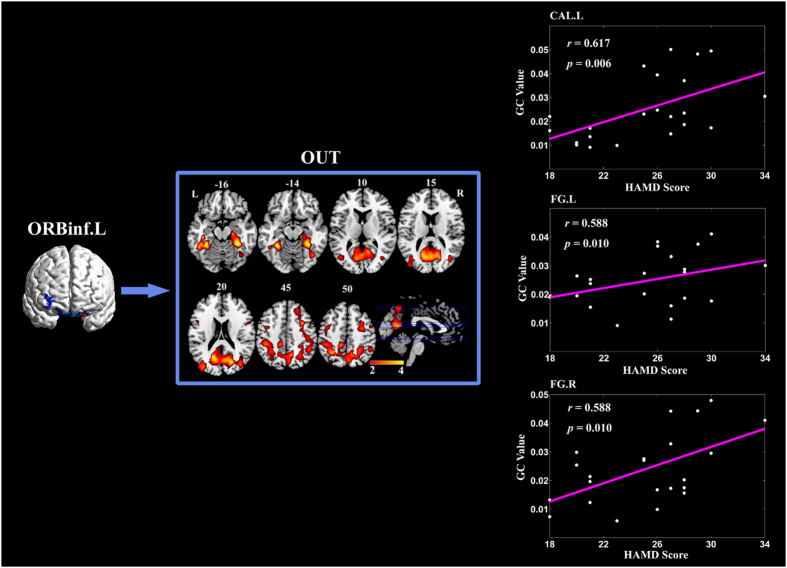
Brain regions with significantly more information transfer from left ORBinf in MDD compared with HC. The median correlation coefficients of the significant correlation coefficients between GC values and patients HAMD scores are shown in the most right column, respectively. Abbreviations: CAL = calcarine fissure, FG = fusiform gyrus, L = left, ORBinf =  inferior frontal gyrus orbital, R = right.

Figure 4 demonstrates the brain regions with significantly higher information transfer from left ORBinf in MDD compared with HC. Regions details are showed in Table 5. MDD patients exhibited significantly higher information transfer from the left ORBinf to the distributed regions including right superior frontal gyrus, bilateral precentral gyrus, superior parietal gyrus, fusiform gyrus, middle occipital gyrus and calcarine fissure. The GC values from left ORBinf to left calcarine fissure, left and right fusiform gyrus have positive correlations with HAMD scores of MDD (See Table 4 for details). Significant difference in the incoming effective connectivity was not found.

### Partially conditioned Granger causality mapping of left CAU seed

**Figure 5:**
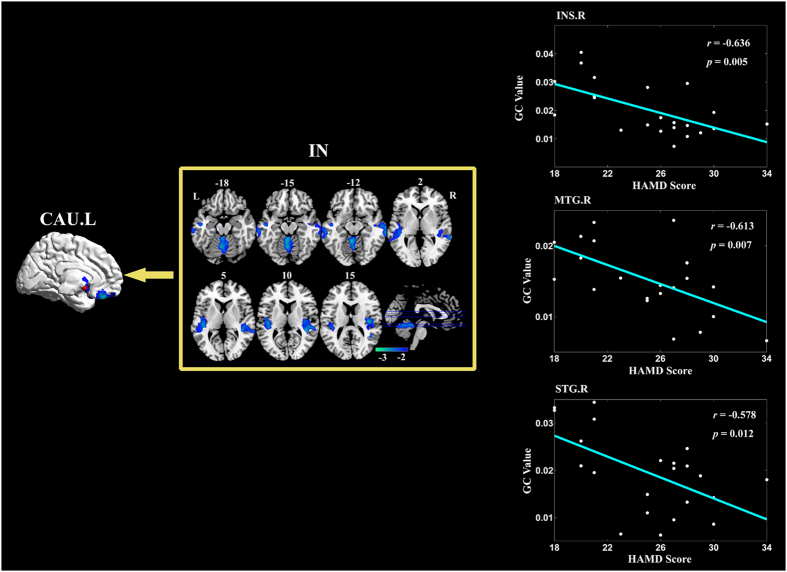
Brain regions with significantly less information transfer to left CAU in MDD compared with HC. The median correlation coefficients of the significant correlation coefficients between GC values and patients HAMD scores are shown in the most right column, respectively. Abbreviations: CAU = caudate nucleus, INS = insula, L = left, MTG = middle temporal gyrus, R = right, STG = superior temporal gyrus.

Figure 5 shows the brain regions with significantly lower effective connections to left CAU for patients comparing to HC group. Significant difference in the outgoing effective connection was not detected. Table 6 summarized the results in details. For the case of left CAU, significantly lower causal inflow was reported from bilateral superior temporal gyrus, middle temporal gyrus, left Heschl gyrus, right INS, and cerebellum_4_5 and vermis_4_5 to the seed. Correlation analysis demonstrated three incoming GC values were negatively correlated with HAMD scores in MDD patients: from right INS, from right middle temporal gyrus and from right superior temporal gyrus (See Table 4 for details).

## Discussion

In this work, we used a blind deconvolution approach to reconstruct the brain HRF at voxel-level to detect the altered HRF shape in first-episode, drug-naïve MDD patients, and further explored the deconvolved BOLD level effective connectivity networks centered at regions with HRF response height abnormalities using partially conditioned GC approach^9^. The method allowed us to explore the spontaneous hemodynamic response shape and to detect the neural causality among brain regions using fMRI data more effectively. Comparing to HC, MDD patients had significantly reduced HRF response height in left ORBsup, right ORBmid, left ORBinf and left CAU. HRF has been found to be related to key physiological factors including cerebral blood flow and the cerebral metabolic rate of oxygen^9^,^19^,^20^,^21^. Especially, this parameter of HRF response height has been found to have striking correlation with cerebral blood flow^21^. Thereby our results suggested that MDD patients likely have decreased brain metabolism and changed cerebrovascular physiology in these regions^20^,^22^.

ORBsup, ORBmid and ORBinf covered the great majority of OFC. OFC and CAU are two critic nodes in affective network, especially in the frontostriatal network^6^,^23^,^24^,^25^,^26^. The frontostriatal network has been identified as having important roles in the regulation and modulation of emotion, behavior and cognition^23^,^24^,^25^,^26^,^27^. OFC, which is also referred to as the ventrolateral prefrontal cortex, is an important area in MDD pathology^27^. This frontal region has a role in the “top-down” and volitional regulation of affect, and is associated with extensive cognitive and affective processes such as decision making, sensory and social information integration, mood regulation and emotional recognition^27^,^28^,^29^,^30^,^31^. Neuroimaging techniques have showed that OFC in patients with MDD have reduced volume^32^, decreased amplitude of low-frequency oscillations^16^, lower biophysical integrity of macromolecular proteins^23^ and lower activation in reward processes^33^. As the dorsal part of striatum, CAU is involved in a range of functions including processing of reward and sensory, the regulation and modulation of affect and mood^23^,^25^. Depressive patients have structural and functional abnormalities in CAU including reduced volumes^34^, decreased regional homogeneity^35^, lower brain metabolism^36^, lower biophysical integrity of macromolecular proteins^23^, aberrant network topological properties^3^,^6^, and lower activation in reward-related processing^37^,^38^. Comparing to HC, significantly lower regional cerebral blood flow were also revealed in the prefrontal cortex and CAU in drug-naïve MDD^39^,^40^,^41^. There has been evidence that reduced regional cerebral blood flow reflects brain metabolism and is related to reduced neuronal size or abnormal vascular factors in these regions^42^,^43^. In the present study, we further demonstrated the decreased height of spontaneous hemodynamic response of OFC and CAU in resting state in MDD. Concerning OFC, the disturbed hemodynamic response may result in the inefficient suppression of maladaptive emotional responses, and may further impair decision making or disrupt the mood regulation and emotional recognition^27^,^28^,^31^. Concerning CAU, the reduced magnitude of hemodynamic response can account for the dysfunction associated with the lack of motivation, ahedonia and altered emotional integration^23^,^37^. Taken together, the decreased magnitude of spontaneous hemodynamic response of OFC and CAU observed in our patients may underlie the disturbances of the emotion-processing circuit, which would contribute to the persistent negative feelings experienced by MDD^16^,^28^. With the HRF parameter of response height, our results provided further evidence for a role of OFC and CAU in the pathophysiology of MDD.

The seed-based GC networks between these regions and the rest of the brain were further computed for MDD and HC. Our results showed significantly stronger information transfer between the seeds in OFC and the distributed brain regions including the frontal, temporal, parietal, occipital and limbic regions in MDD. In addition, the outgoing GC values from right ORBmid to left ACC, and from left ORBinf to left calcarine and bilateral fusiform gyrus were positively correlated with HAMD scores of MDD. Concerning CAU, significantly lower causal inflow was reported from the temporal gyrus, INS and the cerebellum to left CAU in MDD. The incoming GC values from right INS, middle and superior temporal gyrus to the seed were negatively correlated with HAMD scores in MDD. The results demonstrated the causal connectivity disruptions in the brain network associated with cognitive and affective processes. The contrary alterations in the cortico-subcortical network may lead to unbalanced integrating the cognitive and affective information for MDD, and the depressive severity became heavier as the alterations went bigger.

OFC plays a key part in circuit-based models of several psychiatric and neurological disorders because it is extensively connected with diverse neural areas that underlie its participation in a broad range of psychological functions such as processing emotion, reward learning, and decision making^29^,^30^. Previous studies have demonstrated that OFC is not homogenous, but can be parcellated into several cytoarchitectural subregions that possess distinct connections with cortical and subcortical structures^29^,^30^,^44^. In the present study, three subregions in OFC were chosen by comparing the differences of the response height of HRF in the two groups: left ORBsup, right ORBmid and left ORBinf. The three effective connectivity subnetworks centered in the three seeds were further compared between MDD and HC groups.

Chosen left ORBsup as seed, all aberrant outgoing and incoming information transfer for MDD was reported within the frontal network, especially in the dorsal lateral prefrontal cortex. The dorsal lateral prefrontal cortex with its connections to OFC is regarded as the origination point of the volitional/cognitive regulatory arc^31^. Increased functional connectivity has been found between the dorsal lateral prefrontal cortex and OFC in MDD^28^. Our results further demonstrated aberrantly increased forward and backward effective connections between ORBsup and the dorsal lateral prefrontal cortex in MDD, which might affected the modulations of executive control associated with externally induced emotional states in first-episode, drug-naïve MDD patients.

Chosen right ORBmid as seed, MDD patients exhibited stronger effective connections mainly from left PFC and bilateral middle cingulate gyrus to ORBmid. This may lead to improperly modulate the incoming cognitive information in ORBmid for the patients. Stronger outgoing information transfer from right ORBmid to right medial frontal gyrus orbital and left ACC was found. Specifically, the GC values from right ORBmid to left ACC were positively correlated with HAMD scores of MDD patients. ACC, as one of the primary hubs in the salience network, plays a key role in cognitive-emotional integration and ongoing monitoring of behavior^31^. It has been implicated as a focus of dysfunction in depression^5^. Previous functional connectivity studies has found increased connections between ACC and the prefrontal cortex^5^,^45^, indicating the dysfunction in the volitional/external regulatory network for MDD^31^. Our results further demonstrated the abnormal directed connections from ORBmid to ACC, and suggested that ORBmid exhibited increased overdrive of top-down emotional processing to ACC with depression severity in MDD patients.

For the case of ORBinf, there was significant stronger causal outflow to the distributed regions in the frontal, parietal and occipital lobe. The GC values from left ORBinf to left calcarine and bilateral fusiform gyrus had positive correlations with HAMD scores of MDD. These visual processing regions were known to be involved in the perception of emotions in visual stimuli and selective attention^5^,^46^. The disruptions in the resting-state functional connectivity patterns in the visual cortical area for MDD have been reported before, such as aberrant connectivity of the visual cortical areas with regions associated with visual processing^5^,^47^, or abnormal topological property of nodal centrality and nodal degree in the occipital region^3^,^6^. The disruption may contribute to the dysfunction of visual recognition and selective attention in MDD^3^,^5^,^35^. Previous GC analysis demonstrated abnormal effective connectivity in fronto-occipital network^48^. Our results further found the abnormally elevated causal influence from ORBinf to the visual sensory areas, and this aberrant influence increased progressively over the illness severity in MDD. This may contribute to the abnormal top-down effects on the visual cortex during the cognitive information processing from higher cognitive regions to visual sensory regions in MDD^5^,^48^,^49^.

In sum, our study found that OFC have increased abnormal effective connections with a distributed brain regions in first-episode, drug-naïve MDD patients. Specifically, ORBsup demonstrated the aberrant causal connections with the prefrontal cortex. ORBmid has additional abnormal information transfer mainly with the cingulate gyrus; especially the causal outflow to ACC was positively associated with patients’ illness severity. ORBinf showed increased effective connections to the occipital-parietal regions, and the positive correlations were found between the GC values in the visual processing regions and disease severity in MDD. The results demonstrated that different patterns of OFC subnetworks may be associated with specific cognitive and affective processes^29^, and thereby result in differential causal connectivity disruptions in different subregions of OFC in MDD. The further parcellation of OFC in the future OFC-centered network studies will provide better understanding of the psychopathology of MDD.

The effective connectivity mapping reported significantly lower effective connections from the temporal lobe and cerebellum to left CAU for patients comparing to HC group. Incoming GC values from right INS, MTG and STG to the seed were negatively correlated with HAMD scores in MDD patients. The temporal lobe and INS are two critical regions in the affective network^5^,^50^. In addition to primary auditory function, the temporal lobe also plays important roles in social cognition and emotional processing^16^,^50^. INS is regarded as an integration center in bottom-up and top-down information processing including autonomic, visceromotor, emotional, and interceptive information^10^,^51^. The abnormal modulation of the cortical-subcortical loop in the affective network has been observed in MDD^5^,^16^. Our results further indicated decreased causal connectivity from right superior and middle temporal gyrus and INS to left CAU in first-episode, drug-naïve MDD, suggesting inefficient emotional regulation in the cortical-subcortical loop at the early stages of the illness. The abnormal inflow to CAU may result in improperly integrating the incoming information in CAU, and may further worsen the dysfunctional cognitive-emotional processing for the MDD patients^1^. This ability of information integration was found to been worse with the illness severity, for in our study the incoming GC values from the temporal lobe and INS to the CAU had negative correlation with HAMD scores in MDD.

The cerebellum has anatomical connections with the frontal and limbic regions, suggesting that cerebellum contributes to certain emotion and cognitive processing^52^. Abnormalities in the cerebellum in association with depression have been consistently found, indicating the involvement of cerebellar dysfunction in MDD^4^,^5^,^54^,^55^,^56^,^57^. Altered functional connectivity in resting-state has been observed between cerebellum and the default mode network, the executive control network, the limbic and paralimbic regions^5^,^55^,^56^. Specifically, using GC analysis, Guo *et al.* found the inhibitory effect from the temporal gyrus to the cerebellum in MDD^1^. In the present study, we found the decreased inflow from the cerebellum and the temporal gyrus to CAU, suggesting the dysfunction of emotional modulation in the cortico-subcortico-cerebellar network centered around CAU in MDD. The aberrant cerebellar effective connectivity in MDD need to been explored more deeply in the future study.

Interestingly, chosen seeds located in OFC, significantly stronger information transfer were found in MDD; while for the case of left CAU, significantly less information transfer was detected in patients. This suggested hyper-connections of executive control loops centered around OFC which were associated with externally induced emotional states, and hypo-connections of subcortical networks centered around CAU which were related to motivation modulation and emotional integration in first-episode, drug-naïve MDD patients. We speculated that the contrary alterations in the cortico-subcortical network may lead to unbalanced integrating the emotional-related information for MDD, and further exacerbating depressive symptoms.

## Methods

### Participants

Twenty-five first-episode, drug-naïve patients were recruited from the Mental Health Center, West China Hospital of Sichuan University. They all met DSM-IV criteria for MDD according to the diagnostic assessment by the Structured Clinical Interview for DSM-IV-Patient Edition (SCID-P) and were with scores of 18 or greater on the 17-item HAMD. Patients comorbided with other Axis I and Axis II psychiatric disorders such as schizophrenia, bipolar affective disorder, personality disorders and substance abuse or dependence were excluded according to the SCID-I and SCID-II assessment. Twenty-four HC with no history of neurological or psychiatric diseases were also recruited. All subjects were right-handed and without severe or acute medical conditions physically based on clinical evaluations and medical records. The present study protocol was approved by the Ethical Committee of the West China Hospital of Sichuan University and was carried out in accordance with the approved guidelines. The informed written consents were obtained from all participants. There were no significant differences of age, gender, and education between MDD group and HC group. The clinical and demographic data of the recruited subjects were shown in Table 7.

### MRI acquisition

Resting-state fMRI images were acquired using a GE Signa EXCITE 3-T MR system (GE Healthcare, Milwaukee) with an 8-channel phased array head coil. The acquisition parameters were as follows: TR = 2000 ms, TE = 30 ms, Field of view (FOV) = 240 mm, matrix = 64 × 64, voxel size = 3.75 × 3.75 × 5 mm^3^, 30 transverse slices without slice gap, flip angle = 90°, and a total of 205 volumes for each subject. High-resolution T1-weighted anatomical images were also acquired in axial orientation using a 3D spoiled gradient-recalled (SPGR) sequence (TR = 8.5 ms, TE = 3.4 ms, flip angle = 12°, matrix size = 512 × 512 × 156 and voxel size = 0.47 × 0.47 × 1 mm^3^. During the scan, the subjects were instructed simply to rest with their eyes closed, not to think of anything in particular, and not to fall asleep.

### Data preprocessing

Data preprocessing was performed using the Statistical Parametric Mapping software (SPM8, http://www.fil.ion.ucl.ac.uk/spm). The first five volumes were discarded to ensure steady-state longitudinal magnetization. The remaining 200 resting-state fMRI images were first corrected by the acquisition time delay among different slices, and then realigned to the first volume for head-motion correction. The dataset with translational or rotational parameters exceeding ±1.5 mm or ±1.5° would be excluded, remaining the data sets of twenty-two MDD patients and twenty-two HC subjects for further analysis. The images were further spatially normalized into a standard stereotaxic space at 3 × 3 × 3 mm^3^, using the Montreal Neurological Institute template in SPM8. No spatial smoothing was applied in order to avoid artificially introducing local spatial correlation^58^. Since recent studies have showed that functional connectivity analysis is sensitive to gross head motion effects^59^, we further evaluated the framewise displacement (FD) with the suggested threshold of 0.5 to express instantaneous head motion^59^. The largest FD of all subjects was less than 0.2 mm. Two-sample t-test showed there was no significant difference of FD between the two groups (0.057 ± 0.031 for HC and 0.048 ± 0.022 for MDD; *p* = 0.301).Images were then corrected by linear regression to remove the possible spurious variances including six head motion parameters, the white matter, and the ventricular signals averaged from a white matter mask and a ventricular mask respectively. Since removing the global signal may introduce a shift in the distribution of resting-state functional connectivity data and make biological interpretation ambiguous^60^,^61^, we did not remove the global signal in the present study. The residuals of these regressions were temporally band-pass filtered (0.01 < *f* < 0.08 Hz) to reduce low-frequency drifts and physiological high-frequency respiratory and cardiac noise, and linearly detrended for further analysis.

### Partially conditioned Granger causality analysis

In the current study, the partially conditioned GC method in deconvolved BOLD level for effective connectivity analysis was performed using an in-house MATLAB toolbox (http://guorongwu.weebly.com/software.html). Firstly, the voxel-specific HRFs for deconvolution were obtained by a blind deconvolution approach in which resting fMRI was considers as ‘spontaneous events’^9^. These spontaneous events can be detected by point process analysis (PPA), picking up BOLD fluctuations of relatively large amplitude. The HRF for each voxel was then reconstructed by fitting raw BOLD signal using a canonical HRF (two gamma functions) and two derivatives (multivariate Taylor expansion: temporal derivative and dispersion derivative)^9^. Three parameters of the HRF which estimated the potential changes in neuronal activity were calculated including response height, time to peak and full-width at half-max^9^,^62^. The group differences of the three parameters at voxel-level were computed respectively by a two-sample *t*-test in SPM8, to obtain the seeds ROIs for calculating the effective connectivity mapping. Statistical significance was estimated via a Monte Carlo simulation (Alphasim) for multiple comparisons with *p* < 0.05 (single voxel threshold of *p* < 0.04 and cluster size > 190). The REST toolbox (www.restfmri.net) was used to determine the cluster size for Alphasim correction significance, and a brain mask for gray matter was used. ROIs were defined as spheres centered at the local maxima of the statistic *t* map with radius of 6 mm radius.

Then, the causal influence was then calculated between the seeds ROIs and the remaining brain using signals at the neural level by Wiener deconvolution for each subject. The partially conditioned Granger causality approach allows to compute Granger causality conditioned to a limited number of variables in the framework of information theory^14^,^15^. Briefly, consider *n* covariance-stationary variables 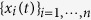, denoting the state vectors as: 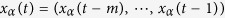, *m* being the model order^9^. The partially conditioned GC component from a variable *β* to another one *α* is defined as follows^15^:


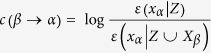


where 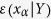 is the mean squared error prediction of 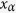 on the basis of the vectors *Y*. 
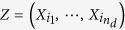
 is a set of the *n*_*d*_ variables, in 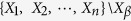, which maximizes the mutual information 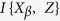
14,15.

Finally, the difference of the effective connectivity networks between MDD and HC groups was further analyzed using two sample *t*-test with age, sex and FD as covariates. The statistically significant threshold was set for multiple comparisons at the cluster level with *p*  < 0.05 using AlphaSim correction (single voxel threshold of *p* < 0.04 and a minimum cluster size of 190 voxels). To investigate whether GC values were correlated with symptom severity and course of disease in MDD patients, relationships between GC values in regions showing significant group differences and HAMD scores/illness duration were further detected by voxel-wise correlation analysis (AlphaSim corrected *p* < 0.05, with single voxel threshold of *p* < 0.04).

## Additional Information

**How to cite this article**: Gao, Q. *et al.* Causal connectivity alterations of cortical-subcortical circuit anchored on reduced hemodynamic response brain regions in first-episode drug-naïve major depressive disorder. *Sci. Rep.* **6**, 21861; doi: 10.1038/srep21861 (2016).

## Figures and Tables

**Table 1:** Summary of the chosen seeds.

Region name	Coordinates						Peak *t*-value
	Abbr.	Hem	X	Y	Z	CS	
Frontal							
Superior frontal gyrus, orbital	ORBsup	L	−18	42	−21	112	−3.04
Middle frontal gyrus, orbital	ORBmid	R	30	57	−6	108	−2.36
Inferior frontal gyrus, orbital	ORBinf	L	−21	24	−24	101	−2.21
Subcortical							
Caudate nucleus	CAU	L	−6	12	−9	95	−2.58

Hem: Hemisphere; CS: Cluster size.

**Table 2:** Brain regions with significantly higher GC values from and to left ORBsup in MDD compared with HC.

Region name	Coordinates						Peak *t*-value
	Abbr.	Hem	X	Y	Z	CS	
Outgoing							
Frontal							
Superior frontal gyrus	SFG	L	−21	54	24	290	3.30
		R	18	33	54	152	2.79
Middle frontal gyrus	MFG	L	−24	36	45	389	2.89
		R	39	9	57	116	3.11
Precentral gyrus	PreCG	L	−51	9	48	62	3.06
	ACC	R	6	39	27	63	3.44
Incoming							
Frontal							
Middle frontal gyrus	MFG	L	−48	12	48	383	3.21
		R	39	51	18	290	3.41
Inferior frontal gyrus, triangular	IFGtri	L	−57	21	30	192	3.76
		R	57	30	0	150	2.90
Inferior frontal gyrus, orbital	ORBinf	R	51	39	−12	82	3.08

Hem: Hemisphere; CS: Cluster size.

**Table 3:** Brain regions with significantly higher GC values from and to right ORBmid in MDD compared with HC.

Region name	C		Coordinates				Peak *t*-value
	Abbr.	Hem	X	Y	Z	CS	
Outgoing							
Frontal							
Medial frontal gyrus, orbital	ORBmed	R	3	51	−3	151	4.72
Limbic							
Anterior cingulate cortex	ACC	L	−12	42	6	117	4.16
Incoming							
Frontal							
Superior frontal gyrus	SFG	L	−18	12	66	161	2.88
Medial frontal gyrus, orbital	ORBmed	L	−48	51	−3	112	3.10
Inferior frontal gyrus, triangular	IFGtri	L	−57	24	18	209	4.04
Inferior frontal gyrus, opercular	IFGoper	L	−60	21	33	93	4.65
Limbic							
Middle temporal gyrus, temporal pole	MTGp	L	−42	9	−27	51	3.21
Middle cingulate gyrus	MCG	L	−9	6	42	104	3.03
		R	9	21	36	44	2.82

Hem: Hemisphere; CS: Cluster size.

**Table 4:** Brain regions with significant correlations between GC values and HAMD scores in MDD patients.

Region name	C		Coordinates				Mean *r*	Std *r*	Mean *p*
	Abbr.	Hem	X	Y	Z	CS			
GC from right ORBmid									
Anterior cingulate cortex	ACC	L	−6	42	6	59	0.557	0.037	0.018
GC from left ORBinf									
Calcarine fissure	CAL	L	−15	−66	18	87	0.619	0.066	0.009
Fusiform gyrus	FG	L	−42	−45	−21	64	0.580	0.052	0.015
		R	27	−39	−15	66	0.572	0.042	0.015
GC to left CAU									
Insula	INS	R	36	−15	15	59	−0.642	0.066	0.007
Middle temporal gyrus	MTG	R	66	−36	−3	102	−0.619	0.063	0.009
Superior temporal gyrus	STG	R	48	−27	9	88	−0.591	0.057	0.013

Hem: Hemisphere; CS: Cluster size.

**Table 5:** Brain regions with significantly higher GC values from left ORBinf in MDD compared with HC.

Region name	C	o	Coordinates				Peak *t*-value
	Abbr.	Hem	X	Y	Z	CS	
Outgoing							
Frontal							
Superior frontal gyrus	SFG	R	18	30	33	149	4.10
Precentral gyrus	PreCG	L	−60	6	39	265	3.11
		R	54	6	33	173	4.01
Parietal							
Superior parietal gyrus	SPG	L	−30	−48	60	218	3.44
		R	21	−51	60	215	4.06
Occipital							
Fusiform gyrus	FG	L	−36	−39	−18	195	4.22
		R	33	−39	−18	186	4.04
Middle occipital gyrus	MOG	L	−39	−84	24	260	3.02
		R	42	−78	27	180	3.21
Calcarine fissure	CAL	L	−12	−69	15	204	3.51
		R	15	−69	18	177	3.27

Hem: Hemisphere; CS: Cluster size.

**Table 6:** Brain regions with significantly lower GC values from left CAU in MDD compared with HC.

Region name	C		Coordinates				Peak *t*-value
	Abbr.	Hem	X	Y	Z	CS	
Incoming							
Temporal							
Superior temporal gyrus	STG	L	−45	−30	9	108	−2.86
		R	42	−33	9	201	−3.50
Middle temporal gyrus	MTG	L	−66	−45	−3	299	−2.69
		R	66	−27	−9	257	−3.11
Heschl gyrus	HES	L	−45	−21	9	46	−2.92
Paralimbic							
Insula	INS	R	39	−18	15	120	−3.03
Cerebellum							
Cerebelum_4_5	CRB_4_5	L	−6	−48	−12	118	−3.05
Vermis_4_5		L	−3	−60	−15	66	−2.89

Hem: Hemisphere; CS: Cluster size.

**Table 7:** Demographics and Clinical Characteristics of the Subjects.

	HC	MDD	*p* value
Age(year)	18–56(36.7 ± 10.9)	18–59(38.4 ± 11.1)	0.615^a^
Gender(male/female)	9/13^b^	9/13^b^	/
Education (year)	6–19(12.1 ± 4.3)	6–19(11.8 ± 4.3)	0.779^a^
HAMD	NA	18–34(25.1 ± 4.3)	/
Illness duration(month)	NA	1–15(7.4 ± 4.3)	/

Data are presented as the range of minimum–maximum (mean ± SD).

HAMD: Hamilton depression scale; HC: Healthy controls; MDD: major depressive disorder; NA: not applicable;

^a^The *p* value was obtained by two-sample *t*-test.

^b^The data of two HC subjects and three MDD patients were excluded because of head motion.
